# Toward a tipping point in federated learning in healthcare and life sciences

**DOI:** 10.1016/j.patter.2024.101077

**Published:** 2024-11-08

**Authors:** Inken Hagestedt, Ian Hales, Eric Boernert, Holger R. Roth, Michael A. Hoeh, Robin Röhm, Ellie Dobson, José Tomás Prieto

**Affiliations:** 1 Apheris AI GmbH, Berlin, Germany; 2 F. Hoffmann-La Roche Ltd., Basel, Switzerland; 3 NVIDIA Corporation, Santa Clara, CA, USA

## Abstract

We discuss the real-world application of federated learning (FL) in the healthcare and life sciences industry, noting a tipping point in its adoption beyond academia. Sharing our experiences with multi-hospital and multi-pharma collaborations, we highlight the importance of involving key stakeholders to develop production-grade FL solutions that are fully compliant with stringent privacy and security standards.

## Main text

The recent federated learning (FL) in digital healthcare collection presents a curation of articles highlighting the use of FL in health contexts. This collection encompasses diverse perspectives, including privacy considerations and research implementations, hinting that we have reached a tipping point in the uptake of FL within healthcare literature. We commend this effort and agree with this main conclusion. In this opinion, we discuss our experiences from real-life applications in the life sciences and healthcare industry, which complement this collection and describe a path toward extending FL beyond academic implementations.

We offer our perspective based on our experience working in real-world initiatives. After the field of FL emerged in 2016,^1^ practitioners tried to apply FL in healthcare setups, but only recently have we seen these solutions supporting repeatable multi-hospital or multi-pharma collaborations: for example, the INTONATE-MS network, a public-private research consortium designed around the privacy-enhancing federated paradigm,^2^ or the AISB Consortium, a multi-pharma collaboration aiming to harness the collective expertise and data of industry leaders to push the boundaries of AI-driven drug discovery.

In this opinion, we first outline our view on which stakeholders should be involved when pursuing a production-grade federation initiative. We then walk through some specific examples of where collaboration between these parties materially results in a solution that goes beyond a federated model—a fully compliant solution that is robust against two key metrics: privacy and security.

### The importance of cross-disciplinary collaboration in FL

FL is poised to improve healthcare; Yang et al.^3^’s recent editorial presents a compelling vision of its potential impact on the healthcare ecosystem. We believe that such benefits can ultimately only be realized via a systematic collaboration among different subject matter experts (SMEs) across a range of disciplines. In particular, the following stakeholders should be involved from the outset in a federated healthcare industrial setting:

(1) Healthcare domain experts: healthcare practitioners who provide relevant use cases, context, and datasets and support the definition of threat models, along with industry partners who control relevant data.
(2) Compliance, security, and privacy experts: legal, privacy, security, and AI governance experts with a focus on ML/FL defining threat models, studying privacy/security attacks, as well as mitigating those attacks.
(3) Federated solution architects and model developers: dedicated solution architects and data scientists with specific knowledge in federated computing are critical to the success of federated initiatives. Many standard software patterns and tools do not apply in a federated setting because of the fundamental requirement of model developers not being allowed to directly access data.

Yang et al.^3^ suggest that without collaboration, separate FL frameworks arise—something readers may infer from the text. However, we want to make this implicit idea clearer: missing any of the above-mentioned groups in a federation initiative can result in outcomes that are inapplicable to real-life scenarios, as described with concrete examples in the following sections.

### A deeper dive into privacy

By its very nature, the data used in healthcare and life sciences model development are often extremely sensitive and bound by strict regulations such as GDPR or the EU AI Act.^4^ Hence, there are legitimate concerns in the healthcare industry that privacy risks can block the adoption of federation as a technology in this sector. In the following, we give some specific examples where a lack of collaboration with dedicated domain and privacy experts can give rise to such risks in the model development life cycle and how these risks can be mitigated.

There is no single privacy measure that can account for all attacks, nor are all attacks universally applicable. Upreti et al.^5^ state that “While studying security, privacy, and trustworthy AI individually, we realized that there is a huge research gap in these fields. We believe that there are strong interconnections and dependencies among these three fields.” The conditions that can make an attack viable will span the domains of AI, federation, healthcare, and information security theory, so it is vital to think through privacy holistically as part of a collaboration with specialist practitioners in those areas, not in isolation.

The effects of such missing collaboration can be observed in Khalil et al.^6^ The authors aim to build a privacy-preserving depression detection model, but the only measure they apply to protect patient privacy *is* FL. It is a common misconception that FL alone is privacy-preserving.^7^ Nevertheless, despite the paper’s valuable insights into the influence of non-i.i.d. data on FL performance, its privacy safeguards are ultimately insufficient.

Clear definitions of threats are essential for choosing the right privacy technologies to mitigate them. We share the surprise and concern that most papers reviewed by Zhang et al.^8^ do not encrypt model parameters sent to the server; a network attacker could directly access the intellectual property (IP)-protected model and infer information about the training data used. However, the use of encryption alone does not solve the problem. A user of the final model could still approximate the model based on its input-output behavior and launch privacy attacks exfiltrating information about the training data used based on the model’s output. Moreover, different types of encryptions have different effects: an encrypted communication channel protects against a network attacker trying to learn model updates directly. If homomorphic encryption is used for the aggregation, the encryption additionally protects against an honest-but-curious server trying to exfiltrate information about the clients’ training datasets at runtime. Encryption technology can be further enhanced by hardware-based solutions, for example by leveraging confidential computing to establish explicit trust among nodes, which further mitigates the possible attack surfaces. To decide which combination of techniques is required, a realistic threat model needs to be defined in collaboration with domain experts.

Pati et al.^9^ provide valuable insights into privacy, but the discussion is not exhaustive, leaving readers mostly unaware of further threats posed by the discussed attacks. Model extraction is not only a loss of confidentiality of the model but also an increase in the vulnerability of the training data to privacy attacks. It also fundamentally threatens the ownership of model IP, which might have been the main commercial motivator to train the model in the first place. Moreover, model poisoning is discussed only in the context of increasing the success of information leakage through other attacks, but poisoning can also lead to poor performance of the model during deployment, limiting the utility of the entire system. They conclude that “[t]here are no turnkey implementations, as solutions require careful configuration [ …] to be effective” which highlights the need of dedicated privacy experts in such FL projects. Additionally, they point out that “[a] significant amount of experimentation on the associated threats and mitigations, crucially in realistic scenarios on real-world data, is required to understand how they play out in different settings where healthcare models are trained using FL,” which also requires collaboration with domain experts and data providers targeting these realistic scenarios.

Privacy in AI is a broadly studied topic, and many of the techniques in the literature apply only under specific conditions. For example, a compromised server can modify the model weights sent to benign clients, exploiting the use of activation functions like ReLU that produce zero-gradient responses for certain inputs. This manipulation allows the server to single out the gradients received from benign clients and thus reconstruct their input data.^10^ This attack is readily mitigated by using a non-flat activation function such as LeakyReLU or by compressing the signal using pooling layers: a common approach in CNNs. Alternatively, a set of malicious clients collaborating to force gradients to zero can increase the information that can be extracted from benign clients.^11^ However, whether compromising a large enough set of clients is feasible is questionable in a realistic setup. Moreover, the use of gradient sharing (federated stochastic gradient descent) is, in general, not recommended due to its inferior privacy protection compared to sharing model weights instead. The above points all demonstrate the importance of realistic use cases and threat models.

Balancing privacy and accuracy of analytical workflows—especially AI models—is often perceived as a zero-sum game, in which favoring one weakens the other.^12^ Giving stakeholders the ability to freely adjust this balance in the FL value stream is pivotal for efficiently reconciling privacy and accuracy. Therefore, practicality and robustness of FL products are necessary in industrial settings. However, practicality cannot introduce additional risks for the healthcare environment. Pharmaceutical companies and hospital settings demonstrate this well. Combining the expertise of security experts, FL specialists, and domain experts in threat modeling exercises makes this possible.

### A deeper dive into security

Security requirements within healthcare data environments such as hospitals and pharma companies are stringent. This is a result of the extreme sensitivity of their data and the regulatory requirements that surround its storage and treatment. Healthcare data custodians such as pharma companies and hospitals have good reason to require strict proof that technology that provides controlled access to their data, as is needed in a federated setting, is safe and compliant.

Research tends to focus on technical approaches to address security, such as model inference attacks or secure aggregation. However, it often neglects architectural fundamentals of distributed systems security in general, and new challenges related to federated solutions in particular. For example, existing rules for defense in-depth and zero-trust architectures are of critical importance in federated collaborations. Other security-related capabilities are often required. For example, penetration testing of products and highly detailed security assessments authored by IT teams are common. Access to a pharma environment may be locked down such that federation vendors are not allowed access to credentials or data locations. Restricted egress and blocked ingress are required for software deployed on the data side. Finally, data custodians may require control down to the computational level over who can do what with their data, and arbitrary code execution must be prevented.

Given the above, it comes as no surprise to us that Zhang et al.^5^ report in their review that no paper provides evidence about the deployment of an FL platform in a real-world healthcare environment. It is necessary to build solutions from the ground up with healthcare-specific security requirements in mind, in collaboration with security experts. However, progress is being made in federated solutions development on this front where industrial federation players are increasingly intensifying their efforts on security. For example, an open-source engine such as NVIDIA FLARE,^13^ when combined with enterprise tooling such as the Apheris Compute Gateway, contains much of the above functionality—restricted egress, traceability, robust authentication, governance at the computational level, and satisfactory penetration testing.

### Conclusion

As noted in Yang et al.,^3^ the adoption of federated computing is flourishing in the healthcare and life sciences industry due to the value it can bring to the management of distributed and highly sensitive data. Building an end-to-end federated computing solution in a data-privacy-critical sector such as healthcare requires collaborative expertise that extends beyond FL algorithm development. Our general recommendation for those seeking to achieve this is to integrate insights from cross-disciplinary SMEs—including model developers; federated solution architects; and security, privacy, and legal experts—from the outset. Though building robust collaborative networks of SMEs may not be trivial, in our experience, this approach significantly reduces the risk of FL initiatives failing to translate into successful real-world applications. Considerations such as commercial ramifications (e.g., model IP), the development of realistic threat models in real-world settings, and the fulfillment of stringent security and compliance requirements from data owners must not be overlooked. These stakeholders will provide the necessary input to build a robust solution from a holistic privacy, security, and compliance perspective.

## Author contributions

Conceptualization, I. Hagestedt, I. Hales, J.T.P., and E.D.; methodology, I. Hagestedt and J.T.P.; writing – original draft, I. Hagestedt, I. Hales, and J.T.P.; writing – review & editing, I. Hagestedt, I. Hales, J.T.P., E.D., R.R., M.A.H., E.B., and H.R.R.

## Declaration of interests

Apheris authors disclose a professional collaboration with Roche and NVIDIA co-authors. E.B. is an employee and shareholder of F. Hoffmann-La Roche Ltd. H.R.R. is an employee and shareholder of NVIDIA Corporation. M.A.H. and R.R. are co-founders of Apheris AI GmbH.
